# Comprehensive Molecular Characterization of Extensively Drug-Resistant *Acinetobacter baumannii* Isolated from Intensive Care Unit Patients: Carbapenemase Genes, Plasmid-Mediated Resistance Determinants, and PFGE-Based Clonal Analysis

**DOI:** 10.3390/ph19060862

**Published:** 2026-05-29

**Authors:** Cihat Öztürk

**Affiliations:** Department of Medical Microbiology, Kırşehir Ahi Evran University, Kırşehir 40100, Turkey; cihat.ozturk@ahievran.edu.tr

**Keywords:** XDR *Acinetobacter baumannii*, PFGE, integrons, OXA genes

## Abstract

**Background**: Colistin- and carbapenem-resistant *Acinetobacter baumannii* (CRAB) represent a critical threat in intensive care unit (ICU) settings. This study aimed to provide a comprehensive molecular epidemiological characterization of extensively drug-resistant (XDR) *A. baumannii* clinical isolates from a tertiary-care hospital in Kırşehir, Central Anatolia, a region previously absent from the national surveillance literature. **Methods**: A total of 43 non-duplicate XDR *A. baumannii* isolates recovered from ICU patients between November 2021 and December 2023 were included. Antimicrobial susceptibility testing was performed by automated systems and broth microdilution for colistin. Resistance genes, including OXA-type carbapenemases, extended-spectrum β-lactamases (ESBLs), metallo-β-lactamases, plasmid-mediated colistin resistance (*mcr-1* to *mcr-5*), plasmid-mediated quinolone resistance genes (*qnr*, *qepA*, *oqxAB*, *aac*(6′)-*Ib-cr*), and class 1 and 2 integrons, were screened by PCR. Integron gene cassettes were characterized by sequencing. Clonal relatedness was assessed by pulsed-field gel electrophoresis (PFGE) using ApaI digestion. **Results**: All 43 isolates exhibited the XDR phenotype with universal resistance to carbapenems, colistin, fluoroquinolones, aminoglycosides (except amikacin), piperacillin, cephalosporins, and tobramycin. Amikacin susceptibility was retained in 58.1% of isolates. *blaOXA-51* was detected in all isolates (100%), and *blaOXA-23* was the predominant acquired carbapenemase (90.7%). Notably, *blaOXA-48*, a carbapenemase typically associated with Enterobacteriaceae, was identified in 3 isolates (7.0%), each belonging to a distinct pulsotype. No *blaOXA-24/40*, *blaOXA-58*, or class B metallo-β-lactamase genes were detected. ESBL genes were found in a subset of isolates, with *blaCTX-M* group 1 being the most prevalent (20.9%). The *aac*(6′)-*Ib-cr* gene was detected in 81.4% of isolates, and *oqxA/oqxB* in 60.5% and 39.5%, respectively. No *mcr* or classical *qnr* genes were identified. Class 1 and 2 integrons were detected in 4.7% and 7.0% of isolates, respectively, carrying *dfrA12*-DUF1010-*aadA2* (class 1) and *dfrA1-sat-1* (class 2) gene cassettes. PFGE identified 12 pulsotypes among the typeable isolates; PT4 (*n* = 20, 47.6%) and PT11 (*n* = 8, 19.0%) were the dominant clonal clusters, together accounting for 65.1% of typeable isolates. **Conclusions**: This study presents one of the first comprehensive molecular epidemiological analyses of XDR *A. baumannii* from Central Anatolia. The dominance of *OXA-23*-carrying clonal lineages, the detection of *blaOXA-48* in *A. baumannii* distributed across three distinct pulsotypes, the high prevalence of *aac*(6′)-*Ib-cr*, and the concurrent distribution of resistance determinants across genetically diverse clonal backgrounds indicate that both clonal expansion and possible horizontal gene transfer contribute to resistance dissemination in this setting. These findings underscore the need for systematic molecular surveillance and reinforced infection control strategies in ICU settings, at both the regional and national levels.

## 1. Introduction

Healthcare-associated infections (HAIs) caused by antimicrobial-resistant bacteria represent one of the most significant global threats to public health. Both the Centers for Disease Control and Prevention (CDC) and the World Health Organization (WHO) have identified HAIs as a major cause of increased morbidity and mortality worldwide [1,2]. It is estimated that HAIs affect approximately 5–15% of hospitalized patients, leading to prolonged hospital stays, severe complications, increased healthcare costs, and excess mortality [1,3]. The progressive limitation of effective antimicrobial treatment options underscores the importance of understanding resistance profiles and underlying molecular resistance mechanisms in order to guide appropriate therapy and reduce the burden of antimicrobial resistance.

A substantial proportion of HAIs are caused by a group of pathogens collectively referred to as the ESKAPE pathogens, an acronym derived from the first letters of *Enterococcus faecium*, *Staphylococcus aureus*, *Klebsiella pneumoniae*, *Acinetobacter baumannii* (*A. baumannii*), *Pseudomonas aeruginosa*, and *Enterobacter* species. These organisms are characterized by their ability to evade antimicrobial activity and rapidly acquire resistance determinants, making them responsible for some of the most difficult-to-treat nosocomial infections [4,5,6].

Among ESKAPE pathogens, *A. baumannii* has emerged as a particularly problematic opportunistic pathogen. Although once considered a low virulence environmental saprophyte with little clinical relevance beyond sporadic opportunistic infections, *A. baumannii* has become a leading cause of severe HAIs due to its remarkable ability to survive for prolonged periods in hospital environments, tolerate a wide range of temperatures and pH conditions, persist on dry surfaces, and spread efficiently between patients [7,8,9,10]. Clinically, *A. baumannii* is frequently isolated from cases of ventilator-associated pneumonia, urinary tract infections, meningitis, wound infections, and bacteremia, particularly in critically ill patients. In addition, community-acquired infections have been reported, especially among individuals with underlying comorbidities [11,12]. Reported mortality rates associated with *A. baumannii* infections range from 7.8% to 23% in hospitalized patients and may reach up to 54% in intensive care units [13,14].

During the 1970s, *A. baumannii* was generally regarded as susceptible to most commonly used antimicrobial agents. However, over the past decades, the organism has demonstrated an extraordinary capacity to acquire resistance, culminating in the emergence of multidrug-resistant (MDR), extensively drug-resistant (XDR), and even pandrug-resistant (PDR) strains. Carbapenems and colistin have long been considered last-line therapeutic options for infections caused by resistant *A. baumannii*; nevertheless, resistance to both agents has increased rapidly in recent years [15,16]. As a consequence, *A. baumannii* has been designated a “red alert” human pathogen [17].

In the WHO priority pathogen list updated in 2024, carbapenem-resistant *A. baumannii* (CRAB) was ranked at the top of the critical priority group, highlighting its status as one of the most urgent global health threats [1]. CRAB infections are associated with high mortality rates and extremely limited therapeutic options, emphasizing the urgent need for detailed molecular epidemiological and resistance mechanism studies [18].

The ability of *A. baumannii* to develop resistance is mediated by multiple mechanisms, including loss or modification of outer membrane porins, production of β-lactamases, overexpression of efflux pumps, antibiotic-modifying enzymes, target site mutations, ribosomal alterations, and modifications of the lipopolysaccharide (LPS) structure [19]. In addition, the *aac*(6′)-*Ib-cr* gene encodes a bifunctional aminoglycoside acetyltransferase variant capable of acetylating both aminoglycosides and fluoroquinolones, particularly ciprofloxacin, via acetylation of its piperazinyl amine, thereby contributing to resistance to both antibiotic classes simultaneously. This gene is plasmid-mediated and represents a clinically significant mechanism of co-resistance to aminoglycosides and fluoroquinolones in Gram-negative pathogens, including *A. baumannii* [20,21,22]. Carbapenem resistance is predominantly associated with OXA-type β-lactamases. Among these, *blaOXA-51*-like enzymes are intrinsic to *A. baumannii* and serve as a species marker, while acquired OXA-type carbapenemases, particularly *blaOXA-23*, *blaOXA-40*, and *blaOXA-58*, confer clinically significant elevated MICs to carbapenems [23,24,25].

Colistin, a cationic lipopeptide antibiotic, exerts its bactericidal effect by interacting with lipid A in the LPS of Gram-negative bacteria and is often regarded as the last-resort antimicrobial for MDR infections [26]. In *A. baumannii*, colistin resistance may arise through multiple mechanisms, including modifications of lipid A mediated by the PmrCAB regulatory system, complete loss of LPS due to mutations in *lpxA*, *lpxC*, or *lpxD* genes, disruption of outer membrane asymmetry, or overexpression of efflux pump systems [16,27,28,29]. Moreover, the discovery of plasmid-mediated mobile colistin resistance (*mcr*) genes in 2015 demonstrated the potential for horizontal transfer of colistin resistance among Gram-negative bacteria. To date, at least ten *mcr* variants (*mcr-1* to *mcr-10*) have been described worldwide [30,31].

This study aimed to comprehensively characterize the molecular epidemiology of extensively drug-resistant (XDR) *A. baumannii* clinical isolates recovered from intensive care unit patients, including the identification of carbapenemase genes, plasmid-mediated resistance determinants, integron gene cassettes, and clonal relatedness by pulsed-field gel electrophoresis (PFGE). A secondary objective was to provide local molecular surveillance data that may contribute to institutional infection control monitoring and epidemiological surveillance efforts in the ICU setting.

## 2. Results

### 2.1. Antimicrobial Susceptibility Profiles

A total of 43 non-duplicate *A. baumannii* isolates were included in the study. All isolates exhibited extensive drug resistance (XDR phenotype). Universal resistance was observed to carbapenems (imipenem), fluoroquinolones (ciprofloxacin and levofloxacin), and colistin. Resistance to gentamicin, piperacillin, cephalosporins, and tobramycin was also observed in all isolates. Resistance to trimethoprim–sulfamethoxazole (SXT) was observed in the majority of isolates (*n* = 35, 81.4%), whereas eight isolates (18.6%) remained susceptible. Partial susceptibility was detected only for amikacin, with 25 isolates (58.1%) remaining susceptible and 18 isolates (41.9%) exhibiting resistance (Table 1).

### 2.2. Distribution of Antimicrobial Resistance Determinants

The intrinsic *blaOXA-51*-like gene, which serves as a species-specific marker for *A. baumannii*, was detected in all isolates (43/43, 100%), consistent with its known presence in this species. Among acquired OXA-type carbapenemase genes, *blaOXA-23* was the predominant determinant, identified in 39 isolates (90.7%). Notably, *blaOXA-48*, a carbapenemase typically associated with *Enterobacteriaceae*, was detected in 3 isolates (7.0%), representing an uncommon finding in *A. baumannii*. No isolates carried *blaOXA-24/40* or *blaOXA-58*. Class B metallo-β-lactamase genes (*blaNDM*, *blaVIM*, and *blaIMP*) were not detected. Additionally, *blaOXA-1* was present in 4 isolates (9.3%) (Supplementary Figure S1).

Regarding extended-spectrum β-lactamase genes, *blaCTX-M* group 1 was identified in 9 isolates (20.9%), *blaCTX-M* group 2 in 1 isolate (2.3%), and *blaSHV* and *blaTEM* in 2 (4.7%) and 3 (7.0%) isolates, respectively. Screening for additional ESBL genes, including *blaGES*, *blaPER*-1, and *blaVEB*, was performed in all isolates; none were detected. Plasmid-mediated pAmpC genes were not detected (Supplementary Figure S2).

All isolates exhibited phenotypic resistance to colistin; however, plasmid-mediated colistin resistance genes (*mcr-1* to *mcr-5*) were absent in all isolates.

Despite universal resistance to ciprofloxacin and levofloxacin, plasmid-mediated quinolone resistance genes (*qnrA*, *qnrB*, *qnrC*, *qnrD*, *qnrS*) and *qepA* were not detected. In contrast, the *oqxA* and *oqxB* efflux pump genes were identified in 26 (60.5%) and 17 (39.5%) isolates, respectively, and the *aac*(6′)-*Ib-cr* gene was detected in 35 isolates (81.4%) (Supplementary Figure S3).

### 2.3. Detection of Integron Gene Cassette

**Figure 1:**
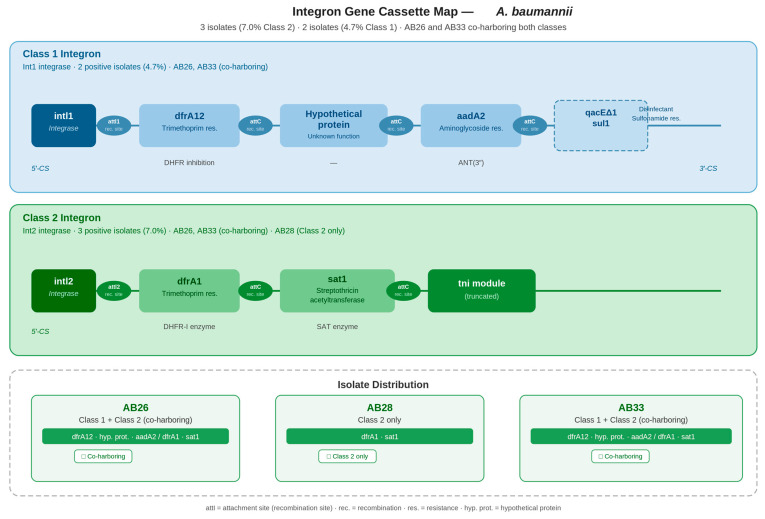
Integron gene cassette map of class 1 and class 2 integrons identified in *A. baumannii* clinical isolates.

In this study, class 1 integrons were detected in 2 isolates (4.7%) and class 2 integrons in 3 isolates (7.0%). All three class 2 integron-positive isolates co-harbored class 1 integrons, resulting in a total of 3 integron-positive isolates. Sequencing of integron gene cassettes revealed the presence of *dfrA12*, *DUF1010* (hypothetical protein), and *aadA2* (Class I), and *dfrA1* and *sat-1* (Class II) gene cassettes (Figure 1).

### 2.4. Pulsed-Field Gel Electrophoresis (PFGE)

**Figure 2:**
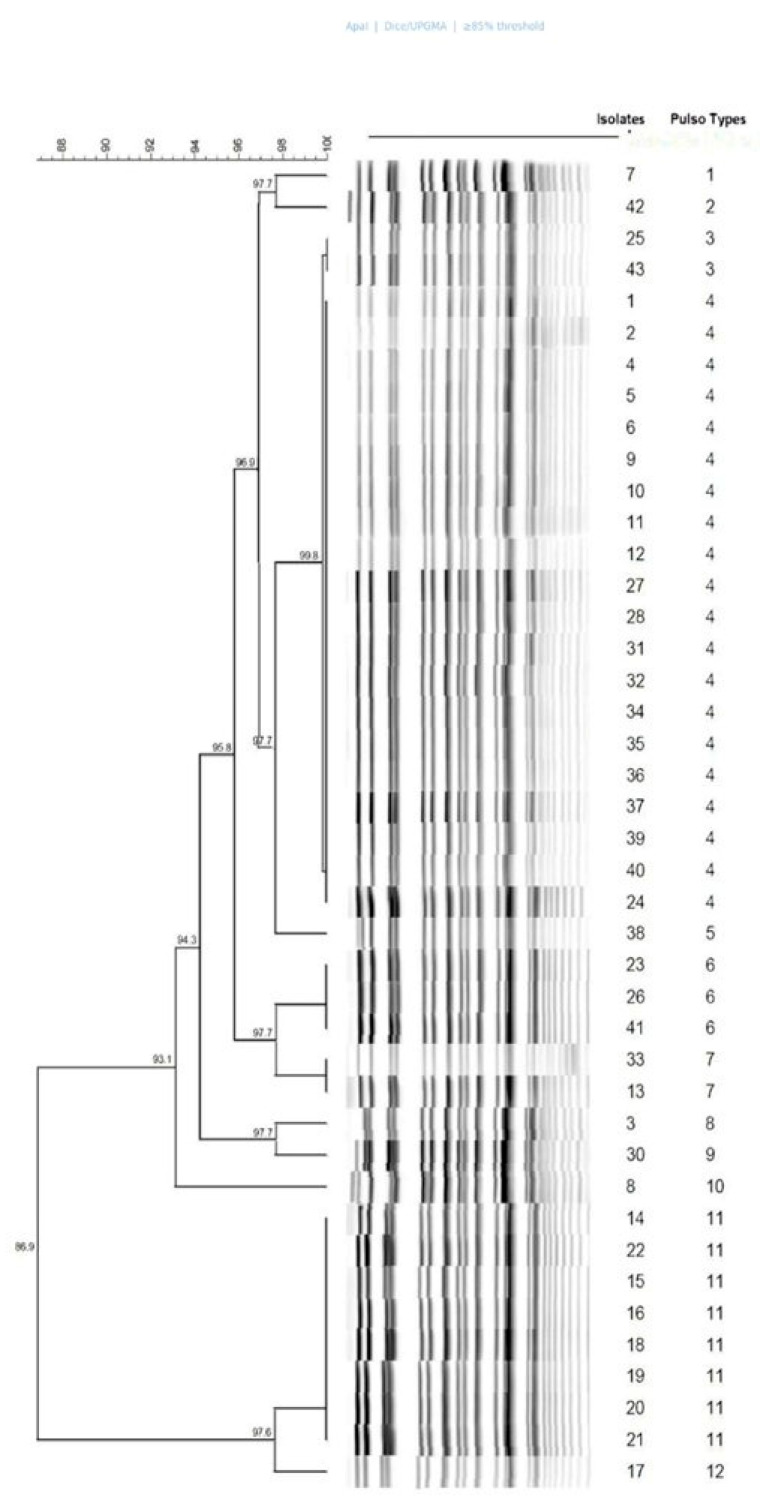
PFGE dendrogram and antimicrobial resistance gene profiles of colistin- and carbapenem-resistant *A. baumannii* clinical isolates. Genomic DNA was digested with ApaI and analyzed using BioNumerics software (version 7.6; Applied Maths, Sint-Martens-Latem, Belgium). Clustering was performed using the unweighted pair group method with arithmetic mean (UPGMA) algorithm with the Dice similarity coefficient. The vertical dashed line indicates the ≥85% similarity threshold used to define distinct pulsotypes. One isolate (AB29) was excluded from analysis due to failure to produce a resolvable banding pattern. Twelve pulsotypes (PT1–PT12) were identified; PT4 (*n* = 20, 47.6%) and PT11 (*n* = 8, 19.0%) represent the dominant clonal clusters.

PFGE analysis was performed on all 43 isolates, all of which were confirmed as *A. baumannii* by *blaOXA-51*-like PCR. One isolate (AB29) failed to produce a resolvable banding pattern and was excluded from pulsotype assignment. Among the 42 typeable isolates, 12 distinct pulsotypes were identified. A dominant pulsotype (PT4) accounted for 20 isolates (47.6%), while the second most frequent pulsotype (PT11) included 8 isolates (19.0%). The remaining isolates were distributed among 10 additional pulsotypes, each representing a minority of cases (Figure 2).

### 2.5. PFGE Analysis in Relation to Antimicrobial Resistance Determinants

Among the 12 identified pulsotypes, PT4 (*n* = 20, 47.6%) represented the dominant clonal cluster. Consistent with the carbapenem resistance data, nearly all PT4 isolates carried bla*OXA-23* in addition to the intrinsic bla*OXA-51* gene, with the exception of one isolate (AB31) that was bla*OXA-23*-negative. Within this cluster, additional β-lactamase genes were infrequently observed. One PT4 isolate (AB28) co-harbored both bla*OXA-23* and bla*OXA-48*. A similar co-occurrence of these two OXA-types carbapenemases was also identified in isolate AB33 belonging to PT7. Integron carriage within PT4 was limited to one isolate: AB28 harbored a class 2 integron encoding dfrA1 and sat-1 gene cassettes.

The second most prevalent cluster, PT11 (*n* = 8, 19.0%), predominantly carried *blaOXA-23* together with the intrinsic *blaOXA-51* gene. No *blaOXA-48* genes or integron-positive isolates were identified within PT11, and ESBL determinants were not observed among isolates belonging to this pulsotype.

Among the typeable *blaOXA-23* negative isolates, AB26, AB30, and AB31 were distributed across distinct pulsotypes. AB26 (PT6) carried *blaOXA-48* as the sole acquired carbapenemase, together with *blaCTX-M* group 1 and 2, *blaSHV*, *blaTEM*, *blaOXA-1*, *oqxA*, *oqxB*, and *aac*(6′)-*Ib-cr*, and harbored both class 1 (*dfrA12*-DUF1010-*aadA2*) and class 2 (*dfrA1*, *sat-1*) integrons, the most complex resistance profile in this study. AB33 (PT7) also co-harbored *blaOXA-23* and *blaOXA-48*, along with both class 1 and class 2 integrons carrying identical gene cassette arrays as AB26. AB30 (PT9) carried only *blaOXA-51* and *oqxA*, without additional resistance determinants. AB31 (PT4), despite belonging to the dominant cluster, lacked *blaOXA-23* and carried only the intrinsic *blaOXA-51* together with *oqxA* and *oqxB*, without additional β-lactamase or integron determinants.

All isolates exhibited phenotypic resistance to colistin; however, *mcr-1–5* genes were not detected in any pulsotype, indicating the absence of plasmid-mediated colistin resistance determinants regardless of clonal lineage.

Plasmid-associated quinolone resistance genes (*oqxA*, *oqxB*, and *aac*(6′)-*Ib-cr*) were identified across multiple pulsotypes, including both dominant and minor clusters, without restriction to a single clonal lineage. Similarly, integron carriage was observed in isolates belonging to PT4, PT6, and PT7, and was not confined to the dominant cluster.

Overall, *blaOXA-23* was predominantly concentrated within the dominant PFGE clusters (PT4 and PT11), whereas other resistance determinants, including *blaOXA*-48, integrons, and plasmid-associated quinolone resistance genes, were distributed across genetically diverse pulsotypes. This distribution pattern may be consistent with the possible contribution of horizontal gene transfer to the dissemination of these resistance determinants.

## 3. Discussion

The present study provides a comprehensive molecular epidemiological characterization of XDR *A. baumannii* clinical isolates recovered exclusively from ICU patients at a tertiary-care hospital in Kırşehir, Central Anatolia. Molecular epidemiological studies addressing the simultaneous characterization of carbapenemase genes, ESBL determinants, plasmid-mediated quinolone resistance genes, integron gene cassettes, and clonal relatedness by PFGE in a single ICU cohort remain scarce in Türkiye, and are particularly rare in Central Anatolia, where systematic surveillance data on *A. baumannii* have been largely absent from the national literature. The present work, therefore, represents one of the few studies from this region to provide such a multidimensional analysis, contributing locally generated data to both the national and international *A. baumannii* surveillance landscape.

All 43 isolates demonstrated the XDR phenotype with universal resistance to carbapenems, fluoroquinolones, colistin, gentamicin, piperacillin, cephalosporins, and tobramycin. This profile closely mirrors the pattern reported by Boral et al. in a prospective multicenter Turkish ICU study, in which all CRAB isolates exhibited comparably extensive resistance profiles and overall patient mortality reached 58.5% [32]. The methodological comparability, both studies drawing exclusively from ICU populations, strengthens the validity of this parallel and reinforces the conclusion that XDR *A. baumannii* has become endemic across ICU settings in Türkiye, including smaller tertiary hospitals in Central Anatolia that have received little previous attention in the surveillance literature. The systematic review by Kılbaş et al. [33], covering national data from 2010 to 2022, confirmed that carbapenem resistance rates in CRAB strains across the country have stabilized above 90%, a threshold our cohort fully reflects.

Partial susceptibility to amikacin was retained in 25 isolates (58.1%), while gentamicin and tobramycin resistance were universal. The *aac*(6′)-*Ib-cr* gene, detected in 35 isolates (81.4%), encodes an enzyme whose substrate preference favors tobramycin and gentamicin over amikacin, which likely underlies this differential susceptibility pattern [34]. The 81.4% prevalence of *aac*(6′)-*Ib-cr* in our cohort exceeds the 65.1% reported by Ghamari et al. [35] in a recent XDR *A. baumannii* ICU cohort from Tehran and the 52.3% rate reported among ciprofloxacin-resistant isolates from Egypt [36]. The higher prevalence observed in our cohort compared to these studies may reflect the sustained selective pressure of combined aminoglycoside and fluoroquinolone use inherent to ICU practice, where broad-spectrum antibiotic exposure is typically prolonged and intensive. The distribution of *aac*(6′)-*Ib-cr* across multiple pulsotypes is consistent with possible dissemination through both clonal expansion and horizontal gene transfer, although plasmid-based transfer was not specifically investigated in this study. The retention of amikacin susceptibility in 58.1% of isolates identifies this agent as the only potentially active aminoglycoside in this collection and underscores the critical importance of strict stewardship policies to preserve its utility for combination regimens in ICU patients with limited therapeutic options.

Among acquired OXA-type carbapenemases, *blaOXA-23* was the predominant determinant, identified in 39 isolates (90.7%). This prevalence exceeds the 67% rate reported by Ahmed et al. [37] in a Turkey–Azerbaijan multicenter study and the 74.4% rate in carbapenem-resistant isolates from the multicenter Turkish study by Boral et al. [32]. The progressive increase in *blaOXA-23* prevalence over time, from the rates reported in earlier studies to the 90.7% observed here, is consistent with the continuing consolidation of this carbapenemase as the dominant acquired resistance determinant in CRAB across Türkiye, a trend confirmed by the systematic review of Kılbaş et al. [33]. The complete absence of *blaOXA-24/40*, *blaOXA-58*, and class B metallo-β-lactamases, in contrast to the *blaOXA-58* predominance documented in earlier Turkish CRAB outbreaks [38], indicates that the carbapenem resistance landscape in our institution is currently defined exclusively by *blaOXA-23*, without the carbapenemase class heterogeneity observed in some other regional settings. The *blaOXA-51*-like gene was present in all isolates (100%), consistent with its role as a species-specific chromosomal marker [25].

The detection of *blaOXA-48* in three isolates (7.0%) is a noteworthy finding that merits careful interpretation. *blaOXA-48* is a carbapenemase whose natural reservoir is Enterobacteriaceae, and its occurrence in *A. baumannii* has been documented only sporadically in the literature. Holden et al. [39] described one such case in a US hospital outbreak in 2021, where whole-genome sequencing provided evidence for interspecies plasmid transfer from *Klebsiella pneumoniae* to *A. baumannii*. In our cohort, the three *blaOXA-48*-positive isolates were assigned to three distinct pulsotypes (PT4, PT6, and PT7). This polyclonal distribution may suggest independent gene acquisition events rather than clonal dissemination, although the underlying transfer mechanism could not be assessed in the absence of plasmid characterization and conjugation experiments, which were beyond the scope of this study. The ICU setting, where co-colonization and polymicrobial infections involving *blaOXA-48* producing Enterobacteriaceae and *A. baumannii* may occur in the same patient or on shared equipment, could represent a facilitating environment for such interspecies gene exchange, though this remains speculative without supporting genomic and epidemiological data. Among the three positive isolates, AB28 (PT4) co-harbored *blaOXA-23* and *blaOXA-48* simultaneously, while AB26 (PT6) carried *blaOXA-48* alongside multiple additional resistance determinants, including ESBL genes, *oqxAB*, *aac*(6′)-*Ib-cr*, and both integron classes. Whether the coexistence of multiple carbapenemase determinants contributes to subtle differences in carbapenem resistance levels compared with *blaOXA-23* only isolates remains to be clarified in future studies with broader quantitative MIC distributions. These observations highlight the importance of routinely screening *A. baumannii* isolates for *blaOXA-48* in ICU settings where *blaOXA-48* producing Enterobacteriaceae are co-circulating, and underscore the need for further genomic studies, including plasmid characterization and whole-genome sequencing, to elucidate the molecular dynamics underlying *blaOXA-48* acquisition in *A. baumannii* in this region.

ESBL genes were detected in a subset of isolates: *blaCTX-M* group 1 was the most prevalent (20.9%), followed by *blaTEM* (7.0%), *blaSHV* (4.7%), and *blaCTX-M* group 2 (2.3%). These rates are broadly comparable to those reported by Boral et al., in which ESBL genes were detected in a minority of CRAB isolates without clustering within specific clonal lineages [32]. The co-detection of ESBL genes alongside carbapenemases and integrons in the same isolates raises concern for the potential horizontal transfer of these determinants to susceptible Enterobacteriaceae co-circulating in the same ICU environment [40], representing an indirect but clinically relevant public health risk.

Universal phenotypic colistin resistance in the absence of *mcr*-1 through *mcr*-5 genes is consistent with the predominant pattern reported in the literature for *A. baumannii*. Zafer et al. [41], in a study of colistin-resistant *A. baumannii* clinical isolates, similarly detected no *mcr* genes and identified *pmrCAB* mutations as the primary chromosomal resistance mechanism. Vijayakumar et al. [42] likewise found no *mcr* variants among 207 *A. baumannii* clinical isolates and confirmed that *pmrAB* mutations were the dominant drivers of colistin resistance. The absence of *mcr* genes in our collection suggests that colistin resistance is likely mediated through chromosomal *pmrCAB* and/or *lpxA/C/D* alterations, consistent with these published reports. The simultaneous resistance to carbapenems and colistin across the entire cohort underscores the critical therapeutic impasse these ICU isolates represent, given that these two agents have constituted the principal last-resort options for CRAB infections. Future whole-genome sequencing studies targeting *pmrCAB* and *lpxA/C/D* loci are warranted to characterize the specific chromosomal basis of colistin resistance in this collection.

Despite universal fluoroquinolone resistance, classical PMQR genes (*qnrA*, *qnrB*, *qnrC*, *qnrD*, *qnrS*, *qepA*) were absent in all isolates. Mohammed et al. [36] similarly reported the absence of *qepA* in a comparable XDR *A. baumannii* cohort and attributed high-level fluoroquinolone resistance primarily to *gyrA*/*parC* target site mutations, consistent with our findings. By contrast, *oqxA* and *oqxB* were detected in 60.5% and 39.5% of isolates, respectively, rates comparable to the 73.3% and 39.5% reported by the same study [36]. The concordance between our *oqxAB* prevalence and that of this geographically distant cohort suggests that *oqxAB* efflux-mediated quinolone resistance is a widely distributed determinant in *A. baumannii* across different regional settings. Its distribution across multiple pulsotypes in our collection is consistent with possible cross-clonal dissemination, though plasmid-based transfer was not directly assessed.

Class 1 integrons were detected in 2 isolates (4.7%) and class 2 integrons in 3 isolates (7.0%), yielding a total of 3 integron-positive isolates, a prevalence notably lower than the high frequencies reported in some regional studies. Khoramrooz et al. [43] detected high frequencies of class 1 and 2 integrons in clinical *A. baumannii* isolates from Iran and identified identical *dfrA12*-DUF1010-*aadA2* and *dfrA1*-*sat-1* gene cassette arrays to those found in our study. The lower integron prevalence in our cohort may reflect the predominantly clonal nature of resistance dissemination in this institution, where *blaOXA-23* appears to have spread mainly through clonal expansion rather than integron-mediated horizontal transfer. Notably, isolates AB26 (PT6) and AB33 (PT7) harbored identical gene cassette arrays despite belonging to distinct pulsotypes, a finding suggestive of possible horizontal integron transfer across genetically unrelated clonal backgrounds, a mechanism well-documented in *A. baumannii* [40] though this interpretation requires confirmation through genomic analysis.

PFGE analysis identified 12 distinct pulsotypes among the 42 typeable isolates, with PT4 (*n* = 20, 47.6%) and PT11 (*n* = 8, 19%) as the dominant clusters, together accounting for 65.1% of all typeable isolates across the full two-year surveillance period. The persistence of PT4 over this entire period, in a cohort comprised exclusively of ICU patients, is consistent with the endemic establishment of this clone in a high-risk environment characterized by prolonged hospital stays, frequent invasive procedures, and intensive broad-spectrum antibiotic use, conditions that create sustained selective pressure and facilitate patient-to-patient transmission. This pattern closely mirrors findings from other Turkish ICU settings: Ertürk et al. [44] reported that 98% of 109 *A. baumannii* ICU isolates from a Turkish university hospital shared highly similar PFGE profiles with *blaOXA-23* as the dominant determinant, and a similar study from a Turkish university hospital found that approximately 80% of *A. baumannii* bloodstream isolates belonged to three dominant genotypes, indicative of sustained cross-transmission in ICU environments [45]. These conditions, well-recognized as drivers of CRAB endemicity in ICU settings, likely underlie the dominance and persistence of PT4 in our institution and warrant a focused epidemiological investigation into transmission routes, including environmental sampling and healthcare worker screening.

Studies combining PFGE with resistance gene analysis in ICU cohorts provide the most directly comparable context for interpreting our pulsotype-level gene distribution. Mohajeri et al., in a PFGE and OXA gene analysis of 75 ICU *A. baumannii* isolates from three hospitals in western Iran, identified four PFGE clusters with two dominant clusters, and reported *blaOXA-23* in 86.5% of isolates, a rate close to our 90.7%, with class 1 and 2 integrons detected in 44% and 36% of pulsotypes, respectively [46]. Notably, in that study, integrons were distributed across multiple pulsotypes, a pattern also observed in our cohort, where the identical integron gene cassette arrays were detected in genetically distinct pulsotypes PT6 and PT7. Ranjbar and Farahani [47], combining PFGE with carbapenemase, MBL, ESBL, and integron gene analysis in 163 Iranian *A. baumannii* isolates, identified 41 PFGE clusters and reported that the most resistant isolates and colistin-resistant strains were concentrated in a subset of dominant PFGE genotypes, with class 1 and 2 integrons detected in 65.6% and 34.4% of isolates, respectively, substantially higher than the 4.7% and 7.0% observed in our cohort. In the Tehran ICU study by Ghamari et al., which combined PFGE with OXA gene typing and integron detection in 55 isolates, 50 distinct pulsotypes were identified, indicating markedly greater clonal diversity than our 12 pulsotypes, and *blaOXA-23* was present in 81.8% of isolates, with class 1 integrons detected in 55.5% of *blaOXA-23* carrying isolates [35]. By contrast, in a PFGE + MLST + integron study of 64 XDR *A. baumannii* ICU isolates from southern Iran, reported three dominant PFGE genotypes all belonging to clonal complex 92, with *blaOXA-23* in 98% of isolates and class 1 integrons in 48%, a clonal dominance pattern more comparable to our PT4-centred distribution, though integron prevalence remained considerably higher than ours [48]. Taken together, these comparisons indicate that while *blaOXA-23* prevalence in our cohort is consistent with or exceeds rates reported in comparable PFGE-based studies from the region, the integron prevalence in our collection is notably lower than most published series, and the degree of clonal dominance, with two pulsotypes accounting for 65.1% of isolates, falls between the highly diverse polyclonal patterns reported in some Iranian ICU series and the near-monoclonal patterns described in others.

## 4. Materials and Methods

### 4.1. Bacterial Isolates and Antimicrobial Susceptibility Testing

The study was approved by the Kırşehir Ahi Evran University Health Sciences Scientific Research Ethics Committee (approval number: 2024-05/22; approval date: 20 February 2024). All procedures were conducted in accordance with the ethical standards of the institutional ethics committee and the principles of the Declaration of Helsinki.

Colistin and carbapenem-resistant A. baumannii isolates were recovered from clinical specimens of patients hospitalized in the intensive care units (ICUs) of Kırşehir Training and Research Hospital, submitted to the Microbiology Laboratory between November 2021 and December 2023. Non-duplicate isolates exhibiting extensively drug-resistant (XDR) phenotypes were included in the study (Supplementary Table S1). Bacterial identification and antimicrobial susceptibility testing were performed using automated systems (VITEK^®^ 2 Compact, bioMérieux, Lyon, France; BD Phoenix™, BD Diagnostic Systems, Franklin Lakes, NJ, USA). Colistin susceptibility was determined by the broth microdilution method, recommended as the reference method by the European Committee on Antimicrobial Susceptibility Testing (EUCAST), and interpreted according to EUCAST criteria [49].

### 4.2. DNA Extraction and PCR Detection of Antimicrobial Resistance Genes

Template DNA was extracted using the boiling method as described by Schlesinger et al. [50]. Conventional PCR was performed using gene-specific primers Supplementary Table S1 to detect β-lactamase genes (Classes A–D), plasmid-mediated colistin resistance genes (*mcr-1–5*), and quinolone resistance determinants.

PCR reactions were performed in a total volume of 25 µL containing 1× PCR buffer, 1.5 mM MgCl_2_, 200 µM of each dNTP, 0.2 µM of each primer, 1 U Taq DNA polymerase, and 5 µL of template DNA (50–100 ng). Amplification conditions were applied according to previously published protocols, and the primer-specific conditions are summarized in Supplementary Table S1. PCR products were analyzed by agarose gel electrophoresis and visualized under ultraviolet illumination.

To confirm the specificity of PCR amplification, representative positive PCR products for each target gene were purified and submitted for bidirectional Sanger sequencing (BMLabosis, Ankara, Turkey). Sequence data were analyzed using NCBI BLAST v2.16.0 (https://blast.ncbi.nlm.nih.gov, accessed on 5 March 2026) against the GenBank database to verify gene identity. Confirmed positive isolates were subsequently used as positive controls in all subsequent PCR reactions for the respective gene targets. Negative controls consisting of sterile deionized water substituted for template DNA were included in each PCR run.

### 4.3. Detection of Integron Gene Cassettes

Detection of class 1 and class 2 integron gene cassettes was performed according to previously described methods [51,52]. PCR amplification of integron gene cassettes was carried out with an initial denaturation step at 95 °C for 3 min, followed by 35 cycles of denaturation at 95 °C for 45 s, annealing at 50 °C for 45 s, and extension at 72 °C for 4 min. A final extension step was applied at 72 °C for 5 min. PCR products were separated by electrophoresis on 1% agarose gels containing ethidium bromide and run at 100 V for 1 h. Gels were visualized under ultraviolet illumination, and the sizes of integron gene cassettes were determined by comparison with a DNA ladder (GolBio Biotechnology Inc., St. Louis, MO, USA). PCR products obtained from integron-positive isolates were sent to BMLabosis (Ankara, Turkey) for DNA sequence analysis.

### 4.4. Pulsed-Field Gel Electrophoresis

Genotypic relatedness among *A. baumannii* isolates was assessed by pulsed-field gel electrophoresis (PFGE) according to the protocol described by Durmaz et al. [53]. Genomic DNA embedded in agarose plugs was digested with ApaI, and electrophoresis was performed using a CHEF-DR III system. PFGE patterns were analyzed using BioNumerics software (version 7.6; Applied Maths, Sint-Martens-Latem, Belgium), and clustering was performed using the Dice coefficient and unweighted pair group method with arithmetic mean (UPGMA), based on Tenover et al.’s [53,54] criteria.

## 5. Conclusions

In conclusion, this study presents one of the few comprehensive molecular epidemiological analyses of XDR *A. baumannii* from Central Anatolia, contributing institutional surveillance data that have been largely absent from both the regional and national literature. The resistance dynamics documented here, including the dominance of *blaOXA-23* carrying clonal lineages, the detection of *blaOXA-48* in *A. baumannii*, and the high prevalence of *aac*(6′)-*Ib-cr*, reflect broader global patterns increasingly reported across diverse ICU settings worldwide. As CRAB continues to be ranked among the highest-priority critical pathogens by the WHO, these findings underscore the need for systematic molecular surveillance, expanded screening for emerging resistance determinants, and coordinated infection control strategies at the institutional, national, and international levels.

## Figures and Tables

**Table 1:** Antimicrobial resistance profiles of colistin- and carbapenem-resistant *A. baumannii* isolates.

Sample Number	Antibiotic Resistance Profile										Antibiotic Resistance Gene Profile											Integrons		
	SXT	GEN	CIP	LEV	IMP	COL	PIP	CAZ	AM	TOB	*CTX M1*	*CTX M2*	*SHV*	*OXA 1*	*OXA 48*	*OXA 23*	*OXA 51*	*TEM*	*oqx A*	*oqx B*	*aac*(6 ′)-*Ib*	Class I	Class II	Integron Gene Profile
AB1	R	R	R	R	R	R	R	R	S	R	−	−	−	−	−	+	+	−	−	−	+	−	−	
AB2	R	R	R	R	R	R	R	R	S	R	−	−	−	−	−	+	+	−	−	−	+	−	−	
AB3	R	R	R	R	R	R	R	R	R	R	−	−	−	−	−	+	+	−	−	−	+	−	−	
AB4	R	R	R	R	R	R	R	R	S	R	−	−	−	−	−	+	+	−	−	−	−	−	−	
AB5	R	R	R	R	R	R	R	R	R	R	−	−	−	−	−	+	+	−	−	−	+	−	−	
AB6	R	R	R	R	R	R	R	R	S	R	−	−	−	−	−	+	+	−	−	−	+	−	−	
AB7	R	R	R	R	R	R	R	R	R	R	−	−	−	−	−	+	+	−	−	−	+	−	−	
AB8	R	R	R	R	R	R	R	R	S	R	−	−	−	−	−	+	+	−	−	−	+	−	−	
AB9	R	R	R	R	R	R	R	R	R	R	−	−	−	−	−	+	+	−	−	−	+	−	−	
AB10	R	R	R	R	R	R	R	R	S	R	−	−	−	−	−	+	+	−	−	−	+	−	−	
AB11	R	R	R	R	R	R	R	R	S	R	−	−	−	−	−	+	+	−	−	−	+	−	−	
AB12	R	R	R	R	R	R	R	R	S	R	−	−	−	−	−	+	+	−	−	−	+	−	−	
AB13	R	R	R	R	R	R	R	R	R	R	−	−	−	−	−	+	+	−	−	−	+	−	−	
AB14	R	R	R	R	R	R	R	R	S	R	−	−	−	−	−	+	+	−	−	−	+	−	−	
AB15	R	R	R	R	R	R	R	R	R	R	−	−	−	−	−	+	+	−	−	−	+	−	−	
AB16	R	R	R	R	R	R	R	R	S	R	−	−	−	−	−	+	+	−	−	−	+	−	−	
AB17	R	R	R	R	R	R	R	R	S	R	−	−	−	−	−	+	+	−	+	−	+	−	−	
AB18	R	R	R	R	R	R	R	R	S	R	−	−	−	−	−	+	+	−	+	−	+	−	−	
AB19	S	R	R	R	R	R	R	R	S	R	−	−	−	−	−	+	+	−	+	−	−	−	−	
AB20	R	R	R	R	R	R	R	R	S	R	−	−	−	−	−	+	+	−	+	+	+	−	−	
AB21	R	R	R	R	R	R	R	R	S	R	−	−	−	−	−	+	+	−	+	+	+	−	−	
AB22	R	R	R	R	R	R	R	R	R	R	−	−	−	−	−	+	+	−	+	−	+	−	−	
AB23	R	R	R	R	R	R	R	R	S	R	+	−	−	−	−	+	+	−	+	+	+	−	−	
AB24	R	R	R	R	R	R	R	R	S	R	−	−	−	−	−	+	+	−	+	+	+	−	−	
AB25	R	R	R	R	R	R	R	R	R	R	−	−	−	−	−	+	+	−	+	+	+	−	−	
AB26	S	R	R	R	R	R	R	R	R	R	+	+	+	+	+	−	+	+	+	+	+	+	+	dfrA12−DUF1010, AadA2 (Class I), dfrA1, sat−1 (Class II)
AB27	R	R	R	R	R	R	R	R	S	R	+	−	−	−	−	+	+		+	+	+	−	−	
AB28	R	R	R	R	R	R	R	R	R	R	+	−	−	+	+	+	+	+	+	+	+	−	+	dfrA1, sat−1 (Class II)
AB29	R	R	R	R	R	R	R	R	S	R	+	−	−	+	−	−	+	−	+	+	−	−	−	
AB30	R	R	R	R	R	R	R	R	R	R	−	−	−	−	−	−	+	−	+	−	−	−	−	
AB31	R	R	R	R	R	R	R	R	S	R	−	−	−	−	−	−	+	−	+	+	−	−	−	
AB32	S	R	R	R	R	R	R	R	S	R	+	−	−	−	−	+	+	−	+	+	+	−	−	
AB33	R	R	R	R	R	R	R	R	S	R	+	−	+	+	+	+	+	+	+	+	+	+	+	dfrA12−DUF1010, AadA2 (Class I), dfrA1, sat−1 (Class II)
AB34	R	R	R	R	R	R	R	R	S	R	−	−	−	−	−	+	+	−	+	−	−	−	−	
AB35	R	R	R	R	R	R	R	R	R	R	+	−	−	−	−	+	+	−	−	+	−	−	−	
AB36	R	R	R	R	R	R	R	R	S	R	−	−	−	−	−	+	+	−	+	+	+	−	−	
AB37	R	R	R	R	R	R	R	R	S	R	−	−	−	−	−	+	+	−	+	+	+	−	−	
AB38	R	R	R	R	R	R	R	R	R	R	−	−	−	−	−	+	+	−	+	+	+	−	−	
AB39	S	R	R	R	R	R	R	R	R	R	−	−	−	−	−	+	+	−	+	−	+	−	−	
AB40	S	R	R	R	R	R	R	R	R	R	+	−	−	−	−	+	+	−	+	+	−	−	−	
AB41	S	R	R	R	R	R	R	R	R	R	−	−	−	−	−	+	+	−	+	−	+	−	−	
AB42	S	R	R	R	R	R	R	R	R	R	−	−	−	−	−	+	+	−	+	−	+	−	−	
AB43	S	R	R	R	R	R	R	R	R	R	−	−	−	−	−	+	+	−	+	−	+	−	−	

AM: amikacin; CAZ: ceftazidime; CIP: ciprofloxacin; COL: colistin; GEN: gentamicin; IMP: imipenem; LEV: levofloxacin; PIP: piperacillin; R: resistant; S: susceptible; SXT: trimethoprim–sulfamethoxazole; TOB: tobramycin; +, gene present; −, gene absent.

## Data Availability

The original contributions presented in this study are included in the article/Supplementary Material. Further inquiries can be directed to the corresponding author.

## Supplementary Materials

The following supporting information can be downloaded at: https://www.mdpi.com/article/10.3390/ph19060862/s1, Table S1. Oligonucleotide primers used for PCR amplification of antimicrobial resistance genes in *A. baumannii*.; Figure S1. Representative agarose gel electrophoresis image showing PCR amplification products of OXA-type carbapenemase genes detected in *A. baumannii* clinical isolates; Figure S2. Representative agarose gel electrophoresis images showing PCR amplification products of extended-spectrum β-lactamase genes detected in *A. baumannii* clinical isolates; Figure S3. Representative agarose gel electrophoresis image showing PCR amplification products of plasmid-mediated quinolone resistance genes detected in *A. baumannii* clinical isolates.
