# Supplementary material for: Comprehensive Molecular Characterization of Extensively Drug-Resistant Acinetobacter baumannii Isolated from Intensive Care Unit Patients: Carbapenemase Genes, Plasmid-Mediated Resistance Determinants, and PFGE-Based Clonal Analysis
Source: Pharmaceuticals (Basel). 2026 May 29;19(6):862. doi: 10.3390/ph19060862 (PMC13306068; doi:10.3390/ph19060862)
Supplement: Supplementary file 1 [file pharmaceuticals-19-00862-s001.zip › pharmaceuticals-4279584-supplementary.pdf]

Supplementary Table S1. Oligonucleotide primers used for PCR amplification of antimicrobial resistance genes in *A. baumannii.*

Primers		5′→3′ Sequences	Amplicon length (bp)	Tm (°C)	Extension time (s)	Referances
TEM		F: AGTATTCAACATTTYCGTGT<br>R: TAATCAGTGAGGCACCTATCTC	847	50	50	(Iraz et al., 2012)
SHV		F: ATGCGTTATATTCGCCTGTG<br>R: TTAGCGTTGCCAGTGCTC	843	56	50	(Hanson et al., 2002)
CTX-M1		F: GCGTGATACCACTTCACCTC<br>R: TGAAGTAAGTGACCAGAATC	260	54	50	(Iraz et al., 2012)
CTX-M2		F: TGATACCACCACGCCGCTC<br>R: TATTGCATCAGAAACCGTGGG	341	55	50	
GES		F: ATGCGCTTCATTCACGCAC<br>R: CTATTTGTCCGTGCTCAGGA	863	57	60	
VEB		F: GATAGGAGTACAGACATATG<br>R: TTTATTCAAATAGTAATTCCACG	914	55	60	(Kiratisin, Apisarnthanarak, Laesripa, & Saifon, 2008)
PER1		F: ATGAATGTCATCACAAAATG<br>R: TCAATCCGGACTCACT	927	48	75	
KPC		F: CGTTCTTGTCTCTCATGGCC<br>R: CCTCGCTGTGCTTGTCATCC	796	58	50	(Iraz et al., 2012)
IMP		F: CATGGTTTGGTGGTTCTTGT<br>R: ATAATTTGGCGGACTTTGGC	488	56	45	
VIM		F: ATTGGTCTATTTGACCGCGTC<br>R: TGCTACTCAACGACTGAGCG	780	59	50	
NDM-1		F: TGGAATTGCCCAATATTATGC<br>R: TCAGCGCAGCTTGTCGGCCATGC	813	58	50	
OXA-1		F: TTTTCTGTTGTTTGGGTTTT<br>R: TTTCTTGGCTTTTATGCTTG	427	50	50	(Coque et al., 2008)
OXA-23		F: GATCGGATTGGAGAACCAGA<br>R: ATTTCTGACCGCATTTCCAT	501	54	45	(Woodford et al., 2006)
OXA-48		F: TTGGTGGCATCGATTATCGG<br>R: GAGCACTTCTTTTGTGATGGC	743	58	50	(Iraz et al., 2012)
OXA-51		F: TAATGCTTTGATCGGCCTTG<br>R: TGGATTGCACTTCATCTTGG	353	56	45	(Woodford et al., 2006)
OXA-58		F: AAGTATTGGGGCTTGTGCTG<br>R: CCCCTCTGCGCTCTACATAC	599	58	50	
OXA-40		F: GGTTAGTTGGCCCCCTTAAA<br>R: AGTTGAGCGAAAAGGGGATT	246	56	30	
pAMPC	*MOX*	F: GCTGCTCAAGGAGCACAGGAT<br>R: CACATTGACATAGGTGTGGTGC	520	64	30	(Altaee & Al-Charrakh, 2023)
	CIT	F: TGGCCAGAACTGACAGGCAAA<br>R: TTTCTCCTGAACGTGGCTGGC	462			
	DHA	F: AACTTTCACAGGTGTGCTGGGT<br>R: CCGTACGCATACTGGCTTTGC	405			
	ACC	F: AACAGCCTCAGCAGCCGGTTA<br>R: TTCGCCGCAATCATCCCTAGC	346			
	EBC	F: TCGGTAAAGCCGATGTTGCGG<br>R: CTTCCACTGCGGCTGCCAGTT	302			
	FOX	F: AACATGGGGTATCAGGGAGATG<br>R: CAAAGCGCGTAACCG GAT TGG	190			
mcr-1		F:CGTTCAGCAGTCATTATGCCAGTTTCTTTCGCGTGC<br>R:CTTACGCATATCAGGCTTGGTTGCTTGTACCGC	320	58	30	(Fan et al., 2022)
mcr-2		F: TGTTGCTTGTGCCGATTGGA<br>R: AGATGGTATTGTTGGTTGCTG	715	52	50	(Chan et al., 2018)

mcr-3	F: CGCTTATGTTCTTTTTGGCACTGTATT<br>R: TGAGCAATTTCACTATCGAGGTCTTG	929	54	50	(Zhang, Wang, et al., 2018)
mcr-4	F: AATTGTCGTGGGAAAAGCCGC<br>R: CTGCTGACTGGGCTATTACCGTCAT	1116	54	50	(Zhang, Chen, et al., 2018)
mcr-5	F: GTGAAACAGGTGATCGTGACTTACCG<br>R: CGTGCTTTACACCGATCATGTGCT	1644	58	120	
qnrA	F: GCCCGCTTCTACAATCAAGT<br>R: GGCAGCACTATTACTCCCAAG	347	59	45	(Hamed et al., 2018)
qnrB	F: TGGCAAAAAATT(GA)ACAGAA<br>R: GAGCAACGATCGCCTGGTAG	594	56	50	(Bouchakour et al., 2010)
qnrC	F: GGGTTGTACATTTATTGAATC<br>R: TCCACTTTACGAGGTTCT	447	56	50	(Wang et al., 2009)
qnrD	F: CGAGATCAATTTACGGGGAATA<br>R: AACAAGCTGAAGCGCCTG	582	57	60	(Chen et al., 2012)
qnrS	F: TCGGCACCACAACTTTTCAC<br>R: TCACACGCACGGAACTCTAT	255	57	30	(Hamed et al., 2018)
qepA	F: CTGCAGGTACTGCGTCATG<br>R: CGTGTTGCTGGAGTTCTTC	403	59	45	(Goudarzi, Azad, & Seyedjavadi, 2015)
oqxA	F: GACAGCGTCGCACAGAATG<br>R: GGAGACGAGGTTGGTATGGA	339	59	30	(Abdalh, Öztürk, Sevim, & Akyol, 2025)
oqxB	F: CGAAGAAAGACCTCCCTACCC<br>R: CGCCGCCAATGAGATACA	240	59	30	
aac(6′)-Ib-cr	F: TTGCGATGCTCTATGAGTGGCTA<br>R: CTCGAATGCCTGGCGTGTTT	482	59	60	(Park et al., 2006)
Class-I Integron	5′-CS-GGCATCCAAGCAGCAAG<br>3′-CS-AAGCAGACTTGACCTGA	Değişken büyüklükte	50	240	(Öztürk, Akyol, Küçükgünay, & Sevim, 2025)
Class-II Integron	hep51: GATGCCATCGCAAGTACGAG<br>hep74: GGGATCCCGGACGGATGCACGATTTGTA	Değişken büyüklükte	50		

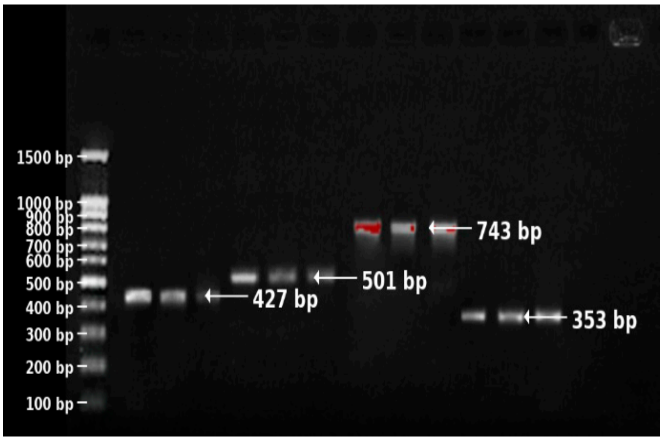

Supplementary Figure S1. Representative agarose gel electrophoresis image showing PCR amplification products of OXA-type carbapenemase genes detected in *Acinetobacter baumannii* clinical isolates. Lane 1: 100 bp DNA ladder; Lane 2: positive control (PK), Lanes 3–4: *bla*OXA-1-positive clinical isolates (427 bp); Lane 5: positive control (PK), Lanes 6–7: *bla*OXA-23-positive clinical isolates (501 bp); Lane 8: positive control (PK), Lanes 9–10: *bla*OXA-48-positive clinical isolates (743 bp); Lane 11: positive control (PK), Lanes 12–13: *bla*OXA-51-positive clinical isolates (353 bp). PCR products were separated on 1% agarose gel stained with ethidium bromide and visualized under ultraviolet illumination. PK: Positive control.

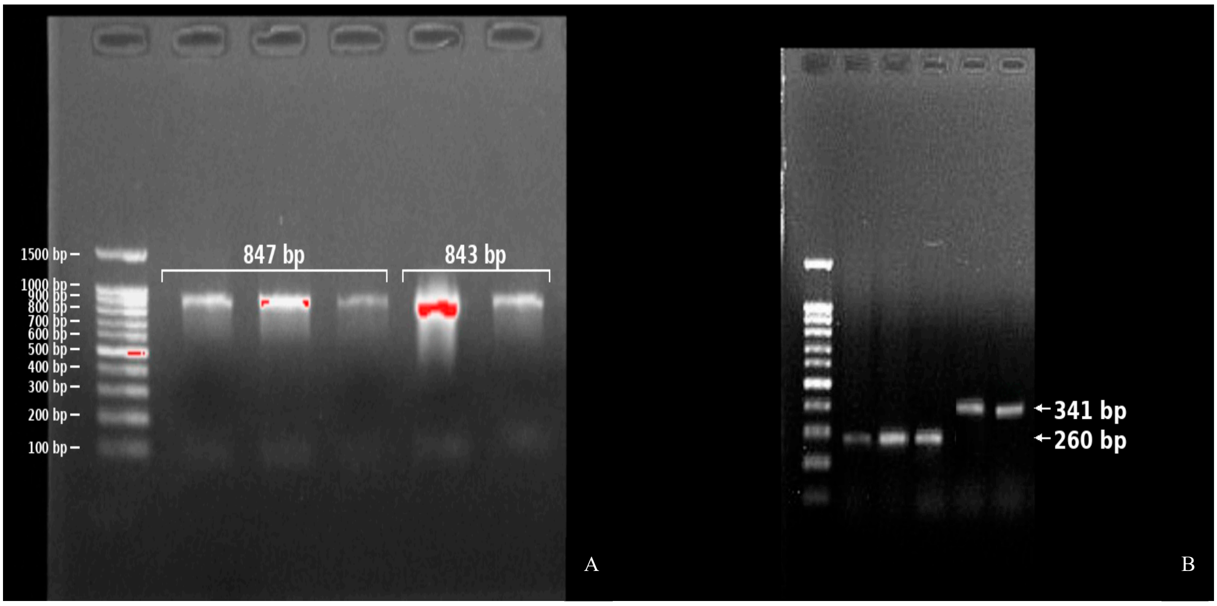

Supplementary Figure S2. Representative agarose gel electrophoresis images showing PCR amplification products of extended-spectrum β-lactamase genes detected in *Acinetobacter baumannii* clinical isolates. **(A)** Lane 1: 100 bp DNA ladder; Lane 2: positive control (PK), Lanes 3–4: *bla*TEM-positive clinical isolates (847 bp); Lane 5: positive control (PK), Lane 6: *bla*SHV-positive clinical isolate (843 bp). (B) Lane 1: 100 bp DNA ladder; Lane 2: positive control (PK); Lanes 3–4: *bla*CTX-M group 1-positive clinical isolates (260 bp); Lane 5: positive control (PK); Lane 6: *bla*CTX-M group 2-positive clinical isolate (341 bp).

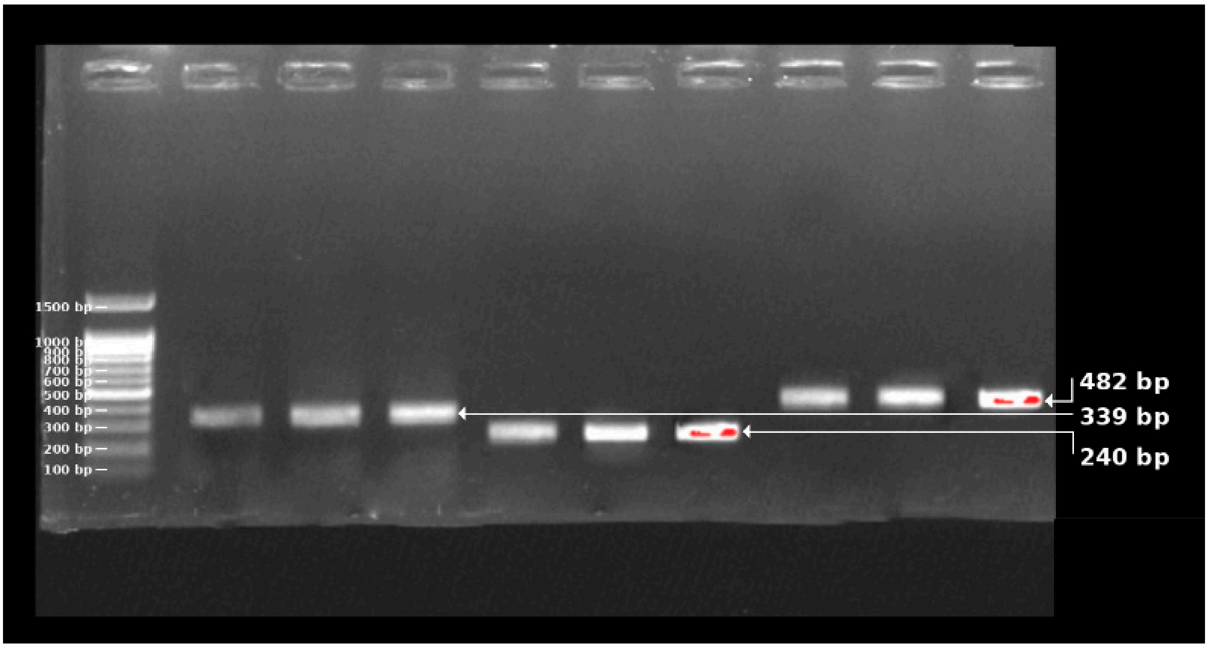

Supplementary Figure S3. Representative agarose gel electrophoresis image showing PCR amplification products of plasmid-mediated quinolone resistance genes detected in *Acinetobacter baumannii* clinical isolates. Lane 1: 100 bp DNA ladder; Lane 2: positive control (PK), Lanes 3–4: *oqxA*-positive clinical isolates (339 bp); Lane 5: positive control (PK), Lanes 6–7: *oqxB*-positive clinical isolates (240 bp); Lane 8: positive control (PK), Lanes 9–10: *aac*(6')-*Ib-cr*-positive clinical isolates (482 bp). PCR products were separated on 1% agarose gel stained with ethidium bromide and visualized under ultraviolet illumination. PK: Positive control.
